# An Analysis of the Digestive and Reproductive Tract Microbiota in Infertile Women with Obesity

**DOI:** 10.3390/ijms252312600

**Published:** 2024-11-23

**Authors:** Jose Bellver, Marta Gonzalez-Monfort, Sandra González, Bruno Toson, Elena Labarta, Gemma Castillón, Giulia Mariani, Carmina Vidal, Juan Giles, Fabio Cruz, Agustin Ballesteros, Marcos Ferrando, Juan Antonio García-Velasco, Diana Valbuena, Felipe Vilella, Marcos Parras-Molto, Esther Tercero-Atencia, Carlos Simon, Inmaculada Moreno

**Affiliations:** 1 IVIRMA Valencia, Pl. de la Policia Local, 3, 46015 Valencia, Spain; 2 Department of Pediatrics, Obstetrics and Gynecology, University of Valencia, Av. de Blasco Ibáñez, 15, 46010 Valencia, Spain; 3 IVI Foundation, Health Research Institute La Fe, Av. Fernando Abril Martorell, 106-Torre A, 46026 Valencia, Spain; 4 Carlos Simon Foundation-INCLIVA Health Research Institute, Ronda Narcis Monturiol Estarriol, 11C, 46980 Paterna, Valencia, Spain; 5 IVIRMA Bilbao, Landabarri Bidea, 3, 48940 Leioa, Biscay, Spain; 6 IVIRMA Barcelona, Carrer Mallorca, 45, 08029 Barcelona, Spain; 7 IVIRMA Madrid, Av. del Talgo, 68, 28023 Madrid, Spain; 8 Igenomix R&D, Ronda Narcis Monturiol Estarriol, 11B, 46980 Paterna, Valencia, Spain; 9 Department of Obstetrics and Gynecology, Beth Israel Deaconess Medical Center, Harvard University, 330 Brookline Ave, Boston, MA 02215, USA

**Keywords:** obesity, female infertility, gut microbiota, endometrium, reproductive tract microbiota, *Streptococcus*

## Abstract

Previous studies have linked the microbiome of distinct body habitats to obesity and infertility; however, the often-divergent results observed have left the role of the so-called “second genome” in obese infertile patients incompletely explored. Here, we present a prospective observational multicenter study of oral, gut, endometrial, and vaginal microbiota of infertile patients classified according to BMI. Patients collected saliva/fecal samples, while vaginal/endometrial fluid samples were collected in the clinic. Total bacterial DNA was extracted, and microbiota profiles were analyzed by 16S rRNA gene sequencing. Our results showed no differences in the Bacteroidetes/Firmicutes ratio (proposed obesity hallmark) in the gut microbiota between patients with obesity and normal weight; however, a tendency for higher levels of genera such as *Escherichia-Shigella* in normal-weight patients was observed; in comparison, patients with obesity possessed increased numbers of *Parasutterella* and *Roseburia*. In the reproductive tract, vaginal samples possessed a similar microbiota to endometrial fluid, both largely colonised by *Lactobacillus*, *Gardnerella*, and *Streptococcus*, supporting the hypothesis that uterine colonisation proceeds from vaginal bacteria ascension. Additionally, higher prevalence of a *Streptococcus*-dominated (>50%) endometrial microbiota was observed among patients with obesity. This first description of the human digestive and reproductive tract microbiota in infertile women with obesity may explain their poor reproductive outcomes.

## 1. Introduction

Obesity affects a significant proportion of women of childbearing age, associates with an increased risk of subfertility/infertility [1,2], and impairs implantation, pregnancy, and live-birth rates during assisted reproductive technologies (ART) [3,4] even with donated ova [5,6].

The intestinal microbiome stimulates development by maintaining epithelial turnover, modulating immune responses, and protecting against pathogens [7]; however, intestinal bacteria also play roles in host nutritional health by regulating micronutrient synthesis and fermentation [8,9] and determining nutrient absorption, and so may contribute to metabolic disorders [10].

Studying gut microbiota composition and associations with body weight in mice and humans provides heterogeneous results [11,12]; meanwhile, evaluations of faecal microbiota transplantation as a treatment for various pathologies have provided a range of clinical responses [13]. Recent studies reported a negative correlation between obesity in females and endometrial receptivity, suggesting that an increasing body mass index (BMI) negatively impacts embryo implantation and the initial stages of pregnancy via reduced uterine receptivity [5,6]. Furthermore, obesity may deregulate endometrial gene expression during the implantation window [14,15,16].

Bacteria in the urogenital tract make up 9% of the human microbiota [17], with vaginal microbiota composed mainly of Lactobacilli [18]. Our laboratory evaluated the endometrial microbiota in infertile patients undergoing in vitro fertilisation (IVF) and associated compositional alterations with reproductive outcomes [19,20]. Our prospective observational study linked an enrichment of *Lactobacillus* with a successful outcome; conversely, an increased abundance of certain bacteria (e.g., *Atopobium*, *Bifidobacterium*, *Gardnerella*, and *Klebsiella*) associated with poorer outcomes [19].

A body of evidence highlights the complex interplay between the microbiota of different body sites, where alterations in one microbiomal habitat can affect distant habitats. For instance, intestinal dysbiosis may contribute directly to colorectal cancer but also indirectly influence the pathophysiology of gynecological cancers/disorders [21]. This effect may be mediated via the production of specific metabolites (e.g., short-chain fatty acids or amino acids) or endotoxins (e.g., lipopolysaccharides) or by modulating free oestrogen levels. Notably, inflammation, obesity, and oestrogen imbalance—recognized risk factors for endometrial cancer—have close links to alterations in the microbiota of the intestinal and reproductive tracts [22]. A deep understanding of how digestive tract microbiota composition influences urogenital tract composition and impacts fertility is still lacking. Studies of the vaginal/cervical microflora in obesity models reported the differential abundance of genera such as *Prevotella* [22], *Lactobacillus* [23,24], *Dialister*, and *Anaerococcus* [22]. The implication of *Fusobacterium* (common in the digestive tract) in the development of ovarian endometriosis suggests crosstalk between the microbiota of distinct body sites [25].

A more in-depth understanding of the microbiota specific to a body habitat in infertile patients with obesity may reveal clinically relevant community alterations. In this work, we explored this concept by mapping the composition of bacterial communities present in several bodily sites—the mouth, gut, vagina, and endometrium—in obese infertile women compared with normal-weight infertile women. For the first time, the microbiota compositions of the oral cavity, gut, uterine cavity, and vagina were described in infertile patients with increasing BMI values. The subtle variations in the bacteria identified may now help to fully define the links between the microbiome and obesity and reveal why women suffering from obesity may suffer from poorer reproductive outcomes.

## 2. Results

### 2.1. Microbiota Diversity and Composition in Infertile Patients with Obesity

The 83 infertile patients recruited were divided into four groups according to BMI. No significant differences were observed between groups when comparing demographic and clinical information (e.g., age, indication for ART, obstetric formula, and concomitant medication) (Table 1).

**Figure 1:**
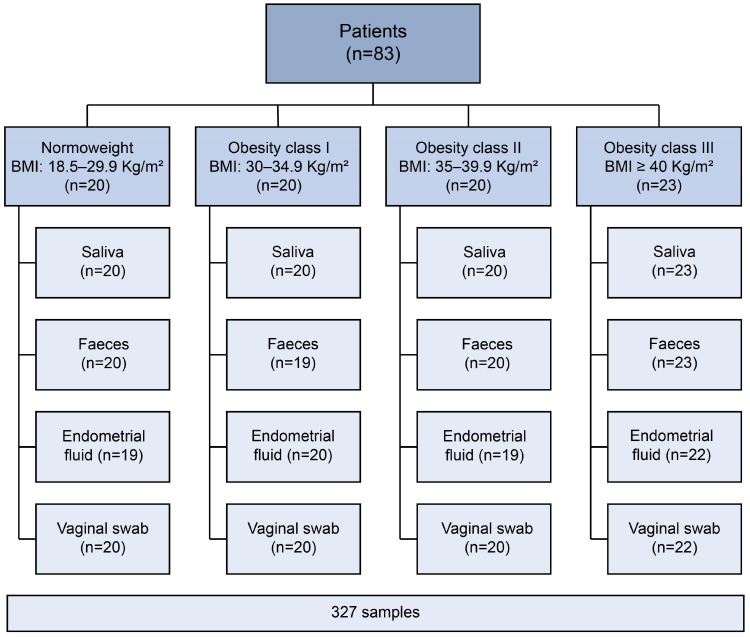
Schematic view of collected samples. Eighty-three patients from four centres were placed in four groups according to BMI values. A total of 327 samples of saliva, faeces, endometrial fluid, and vaginal swabs were used to describe the human digestive and reproductive tract microbiota in infertile patients with obesity and to assess any relationship between the microbiota and obesity.

The bacterial microbiota of the oral cavity, gut, endometrium, and vagina were profiled by 16S rRNA gene sequencing of 327 SA, FE, EF, and VA samples (Figure 1), finding values (and range) for the mean total sequencing reads per sample of 219,659 in SA (13,682–869,467), 217,565 in FE (7414–861,544), 228,056 in EF (34,451–1,577,473), and 243,948 in VA (28,899–934,367) samples. After quality control filtering, reads were mapped to bacterial databases to obtain a taxonomical assignment as operational taxonomic units (OTUs) and were transformed into clr data.

**Figure 2:**
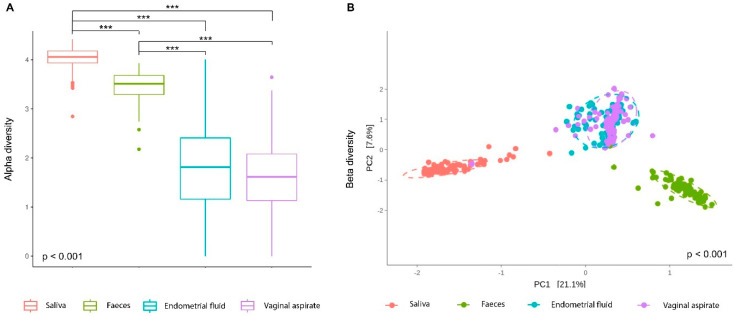
Alpha and beta diversities of the human microbiota in infertile patients with normal weight and obesity. (**A**) Alpha diversity (Shannon index) of body habitats. Comparisons between sample types were performed using Kruskal–Wallis, and two-by-two comparisons were performed using the Dunn test. (**B**) Principal component analysis (PCA) plot of beta diversity after PERMANOVA test for both general and two-by-two comparisons. *** *p* < 0.001.

Shannon index values (alpha diversity estimator) suggested that SA samples possessed the most complex microbiota structure (higher values) followed by FE samples, which displayed a similar level of complexity; in contrast, bacterial communities in EF and VA samples displayed a similar, simpler structure (Figure 2A). Significant differences were found in alpha diversity between oral, gut, and reproductive tract microbiota when using two-by-two comparisons (*p* < 0.001); however, no differences were found within the reproductive tract itself (EF and VA, *p* = 0.965). Principal component analysis (PCA) of beta diversity demonstrated significant differences when comparing all samples (*p* < 0.001). In agreement with alpha diversity results, broad similarities were found between the microbiota of EF and VA samples, whereas SA and FE samples possessed distinct community structures when compared with any sample type individually (*p* = 0.006 for all comparisons) (Figure 2B).

These results report the digestive and reproductive tract microbiota in infertile women with obesity.

#### 2.1.1. Microbiota of the Oral Cavity in Infertile Patients with Obesity

**Figure 3:**
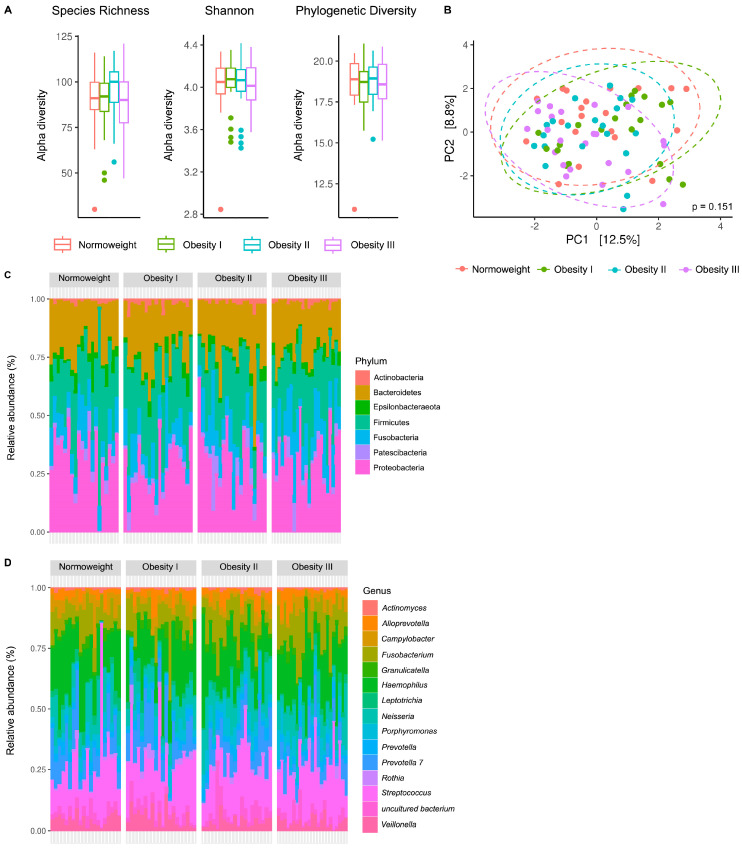
Microbiota composition of the oral cavity in infertile patients with normal weight and obesity. (**A**) Differences in alpha diversity (Species Richness, Shannon, and Phylogenetic Diversity indexes) was analysed using the ANOVA test. (**B**) Principal component analysis (PCA) of beta diversity after PERMANOVA analysis. (**C**,**D**) Microbiota structure of saliva samples at the (**C**) phylum and (**D**) genus levels. Normal weight (BMI: 18.5–29.9 Kg/m^2^), Obesity I (BMI: 30–34.9 Kg/m^2^), Obesity II (BMI: 35–39.9 Kg/m^2^), Obesity III (BMI ≥ 40 Kg/m^2^).

The alpha diversity of SA samples was assessed through a Species Richness (SR) estimator (OTU richness), a richness and evenness estimator (Shannon index), and a Phylogenetic Diversity (PD) estimator (Figure 3A). Considering all groups, an average SR value of 90 for the oral cavity (OTU count of SA samples) was encountered. The Shannon index estimates sample richness and considers OTU evenness, while the PD estimator considers bacterial phylogeny to estimate diversity across the phylogenic tree. No significant differences among the three obesity and normal weight groups were observed in these alpha diversity measures for the SA microbiota (Figure 3A). No significant differences between patients of differing BMI values were observed when considering the beta diversity of our samples (PCA demonstrates a lack of significant clustering by group), suggesting no relation between BMI values and SA sample microbiota structure (Figure 3B).

Next, SA sample compositions were assessed at the phylum (Figure 3C) and genus (Figure 3D) levels. The oral cavity possessed a rich, balanced microbiota structure with abundant Bacteroidetes, Firmicutes, and Proteobacteria and a less prominent presence of Epsilonbacteraeota, Fusobacteria, and Patescibacteria (Figure 3C). The fifteen most abundant genera were considered in SA samples (Figure 3D and Figure S1A,B depict an average of samples), and a general abundance of *Fusobacterium*, *Haemophilus*, *Neisseria*, *Prevotella* 7, and *Streptococcus* combined with a lower number of genera such as *Alloprevotella*, *Campylobacter*, *Porphyromonas*, and *Veillonella* was observed. Following the trend in Figure 3B, differences were not detected in bacterial composition at the phylum or genus level between patients of differing BMI values.

Our findings agree with reported data regarding the presence of specific genera but provide no evidence for a link between changes to the oral microbiota in infertile patients with normal weight and those with obesity.

#### 2.1.2. Gut Microbiota in Infertile Patients with Obesity

**Figure 4:**
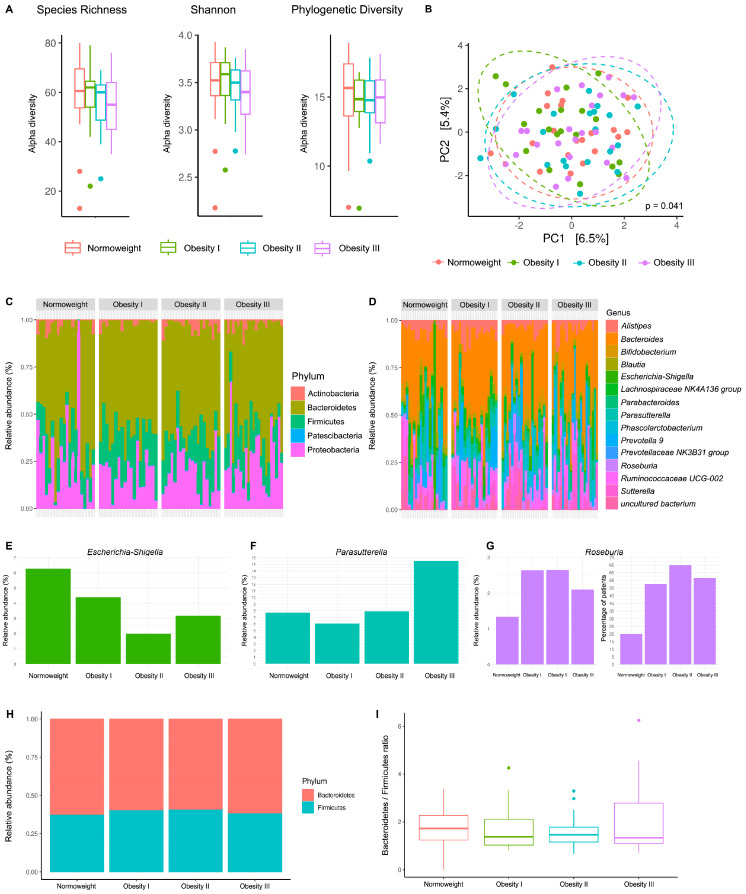
Microbiota compositions of the gut in infertile patients with normal weight and obesity. (**A**) Differences in alpha diversity (Species Richness, Shannon, and Phylogenetic Diversity indexes) were analysed using the ANOVA test. (**B**) Principal component analysis (PCA) of beta diversity after PERMANOVA analysis. (**C**,**D**) Microbiota structure of faecal samples at the (**C**) phylum and (**D**) genus levels. (**E**–**G**) Differential abundance of faecal taxa in normal weight patients and patients with obesity: (**E**) relative abundance of *Escherichia-Shigella*, (**F**) relative abundance of *Parasutterella*, and (**G**) relative abundance and percentage of patients with the presence of *Roseburia*. (**H**,**I**) Bacteroidetes/Firmicutes relative abundance (**H**) and ratio (**I**) in patients classified according to BMI values. Comparisons assessed by ANOVA test. Normal weight (BMI: 18.5–29.9 Kg/m^2^), Obesity I (BMI: 30–34.9 Kg/m^2^), Obesity II (BMI: 35–39.9 Kg/m^2^), Obesity III (BMI ≥ 40 Kg/m^2^).

Compared with an average SR value of 90 for the oral cavity (all patients), the FE samples possessed a lower SR average of 56, suggesting a lower level of alpha diversity than the SA samples (Figure 4A).

Considering all alpha diversity measures, no significant differences were revealed between patients of differing BMI values (Figure 4A); however, a significant difference was observed when considering FE sample beta diversity (*p* = 0.041)—an association between increasing BMI value and microbiota composition (Figure 4B).

FE sample analysis provided evidence of a less diverse community than the SA samples, with Firmicutes and Proteobacteria present at similar levels alongside highly abundant Bacteroidetes; furthermore, Actinobacteria and Patescibacteria were detected at low levels (Figure 4C). At the genus level, FE samples possessed a rich and diverse microbiota, with an abundant presence of *Bacteroides* and additional genera (e.g., *Alistipes*, *Parasutterella*, *Prevotella* 9, and *Sutterella*) (Figure 4D and Figure S1C,D). A small sample size and high within-group variability (F value = 1.23) represent the main limitations when exploring the differential abundances of individual bacteria; overall, specific taxa whose abundance may produce the significant differences found between patients of differing BMI values could not be detected (Figure 4B). However, an increased relative abundance of *Escherichia-Shigella* in normal-weight patients (Figure 4E) and an increased relative abundance of *Parasutterella* among patients with higher BMI values (Ob-III) were detected (Figure 4F). An increased relative abundance of *Roseburia* in patients with obesity and a two-to-three-fold increase in the percentage of *Roseburia* present (over 1%) in the FE samples of patients with obesity were also observed (Figure 4G).

Regarding the current hypothesis of differences in the gut microbiome of patients with obesity or related metabolic diseases, the content of Bacteroidetes (B) and Firmicutes (F) in FE samples represents a debated hallmark of obesity [26]. The B/F ratios for all patients were calculated (Figure 4H), but statistically significant differences were not detected when comparing groups of distinct BMI values (*p* = 0.58) (Figure 4I).

Our results suggest a significant difference in the gut microbiota in infertile patients according to BMI values; however, the bacteria genera responsible for this feature were not identified due to the small sample size and high variability observed between patients. Our findings suggest that the abundance of genera such as *Escherichia-Shigella*, *Parasutterella*, and *Roseburia* relates to obesity, which deserves further investigation.

#### 2.1.3. Reproductive Tract Microbiota in Infertile Patients with Obesity

**Figure 5:**
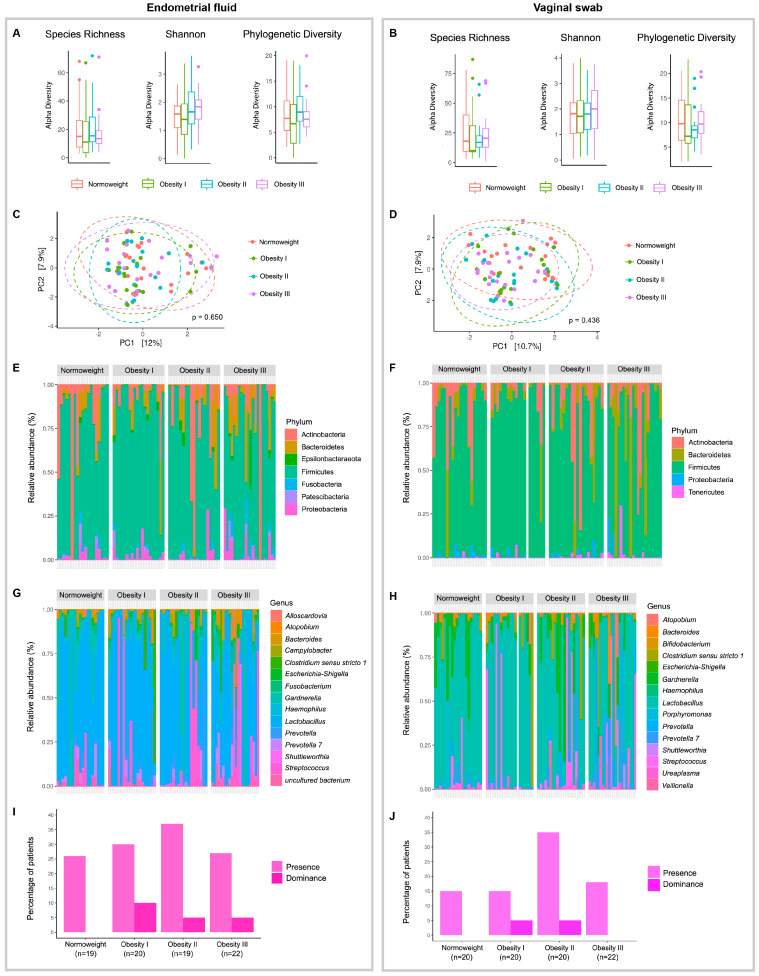
Microbiota composition of the reproductive tract. Analysis of endometrial fluid (**A**,**C**,**E**,**G**,**I**) and vaginal aspirate **(B**,**D**,**F**,**H**,**J**) samples in infertile patients with normal weight and obesity. (**A**,**B**) Differences in alpha diversity (Species Richness, Shannon, and Phylogenetic Diversity indexes) were analysed using the ANOVA test. (**C**,**D**) Principal component analysis (PCA) of beta diversity after PERMANOVA analysis. (**E**–**H**) Microbiota structure of endometrial fluid and vaginal aspirate samples at the (**E**,**F**) phylum and (**G**,**H**) genus levels. (**I**,**J**) Percentage of patients with (**I**) presence (>1%) and (**J**) dominance (>50%) of *Streptococcus* in endometrial fluid and vaginal aspirate samples in each study group. Normal weight (BMI: 18.5–29.9 Kg/m^2^), Obesity I (BMI: 30–34.9 Kg/m^2^), Obesity II (BMI: 35–39.9 Kg/m^2^), Obesity III (BMI ≥ 40 Kg/m^2^).

Next, vaginal and endometrial microbiota were evaluated by analysing EF and VA samples (Figure 5). Low average SR values for EF (23) and VA (19) (Figure 5A,B) compared with SA and FE were found (Figure 3A and Figure 4A), suggesting a lower alpha diversity in the reproductive tract compared with the oral cavity/gut. No significant differences between patients of differing BMI values in any alpha diversity measure (Figure 5A,B) or beta diversity analysis (Figure 5C,D) for the EF or VA samples were observed, respectively, suggesting that increasing BMI values were not associated with vaginal/endometrial dysbiosis.

The EF and VA samples displayed broad similarities at the phylum level, with low levels of Actinobacteria, Bacteroidetes, and Proteobacteria and minimal levels of specific phyla (e.g., Epsilonbacteraeota and Fusobacteria) in a Firmicutes-dominated population (Figure 5E,F). At the genus level, EF and VA samples displayed significant similarities—a *Lactobacillus*-dominated community in the presence of *Gardnerella* and *Streptococcus* and a small number of *Clostridium sensu stricto*, *Prevotella*, and *Veillonella* (Figure 5G,H and Figure S1E–H).

The similarity of the bacterial composition of the EF and VA samples supports the hypothesis that vaginal bacteria ascension leads to endometrial colonisation. Nonetheless, differences in bacterial communities between sample types were present; considering only the fifteen most common genera, *Alloscardovia*, *Campylobacter*, and *Fusobacterium* were only encountered in EF samples and *Bifidobacterium*, *Porphyromonas*, and *Ureaplasma* were only encountered in VA samples. Following the trends in Figure 5C,D, no significant differences in the overall bacterial composition at phylum or genus levels between patients of differing BMI values were detected.

Subclinical reproductive tract infections, including chronic endometritis [27], are linked to higher incidences of recurrent pregnancy loss and repeated implantation failure; therefore, the abundance of chronic endometritis-causing bacteria in EF and VA samples was evaluated. While *Chlamydia*, *Enterococcus*, *Staphylococcus*, and *Mycoplasma* were not detected, *Escherichia-Shigella*, *Gardnerella*, and *Klebsiella* levels remained similar among groups (Table S1). An interesting, although not statistically significant, difference was encountered in *Streptococcus* abundance in EF and VA samples among the groups (Table S1). No differences in the presence (levels above 1%) of *Streptococcus* spp. were found between patients of differing BMI values (Figure 5I,J); nonetheless, dominance (<50%) of *Streptococcus* spp. was only observed in patients with obesity (Figure 5I,J).

Our results agree with previous reports describing the microbiota composition of the reproductive tract. Although not statistically significant, infertile patients with obesity appear more prone to possessing *Streptococcus*-dominated reproductive microbiota, a feature that could shed light on patterns of infertility among patients with obesity.

### 2.2. Analysis of the Human Digestive and Reproductive Tract Microbiota in Infertile Women with Obesity

Additional factors that may cause differences in the microbiota were investigated to confirm the interesting variations observed in the composition of gut and reproductive tract microbiota in infertile patients according to BMI values. After PCA, no significant associations between microbiota composition alterations and infertility cause, the incidence of polycystic ovary syndrome, or age were revealed in our results (Figure S2); thus, factors other than BMI were not encountered that associated with bacterial composition deviation.

**Figure 6:**
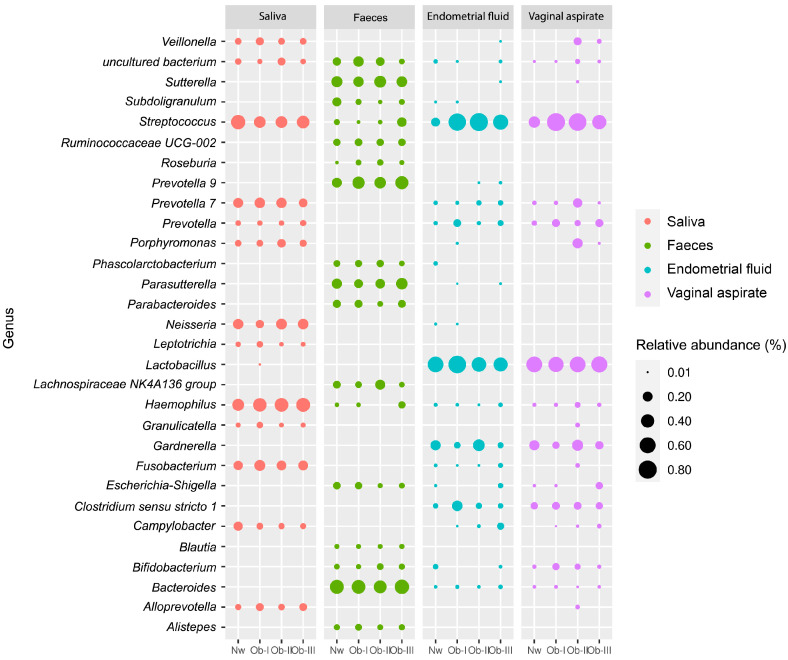
Comparison of microbiota composition between all body habitats. Relative abundance (size) of taxa in the microbiota of infertile patients with obesity in saliva, faeces, endometrial fluid, and vaginal samples. Data for infertile patients with normal weight (Nw) and obesity (Ob-I, Ob-II, Ob-III).

These results help to describe the digestive and reproductive tract microbiota in patients with obesity. Specific signature taxa colonised each body habitat, making their microbiota highly distinct (Figure 6).

Significant amounts of genera such as *Alloprevotella*, *Campylobacter*, *Fusobacterium*, *Haemophilus*, *Neisseria*, *Prevotella* 7, and *Streptococcus* characterised SA samples, while FE samples possessed a microbiota composed of abundant Bacteroides, with significant numbers of *Parabacteroides*, *Parasutterella*, *Prevotella* 9, *Roseburia*, and *Sutterella*. Lastly, reproductive tract samples (EF and VA) displayed a characteristic *Lactobacillus*-dominant microbiota with a significant presence of *Gardnerella* and *Streptococcus*. Although the exclusive presence of specific genera was not observed in a specific body habitat, their abundance correlated with sample type; thus, distinctive bacterial composition profiles comprise the microbiota of distinct body sites.

## 3. Discussion

This study reports the first description of the digestive and reproductive tract microbiota in infertile women with obesity. Although vast differences in microbiota structure were not observed within body sites, women with obesity generally possessed distinct levels of genera such as *Escherichia-Shigella*, *Parasutterella*, and *Roseburia* in the gut and *Streptococcus* in the reproductive tract.

Human microbiome composition within body sites has been linked to pathological conditions such as endometriosis, repeated implantation failure, inflammatory bowel disease, and gingivitis [28,29]. The relationship between our so-called “second genome” and metabolic diseases has been previously studied [11,12]; however, whether dysbiosis explains the poorer reproductive outcomes of patients with obesity remained unknown [5,6,14].

The oral microbiota, among the most diverse in the human body, presents a significantly different bacterial community composition than the gut. The principal bacteria isolated from healthy oral cavities include *Neisseria*, *Streptococcus*, *Prevotella*, and *Veillonella* [29,30,31]. The SA microbiota possessed the highest alpha diversity scores, with a composition in line with previously reported studies—*Neisseria*, *Streptococcus*, *Prevotella*, and *Veillonella* displayed a general abundance in SA samples alongside bacteria such as *Fusobacterium*, *Haemophilus*, and *Porphyromonas* [29,30,31]. Differences in bacterial communities were not detected when considering the patient’s BMI values.

Our findings regarding the gut microbiota also agree with previous reports [11,32], with *Escherichia-Shigella*, *Parasutterella*, and *Sutterella* being commonly found in the FE samples and *Bacteroides* being the most abundant genus. While the influence of gut microbiome composition on obesity/metabolic diseases has been previously evaluated, how changes in bacterial populations induce these pathologies remains unknown. Recent systematic reviews evaluating gut bacteria composition in patients with/without obesity reported a range of results [11,12]. Crovesy et al. reported higher counts of Firmicutes and Proteobacteria in patients with obesity and decreased abundances of Bacteroidetes and Verrucomicrobia [11]. Pinart et al. described lower proportions of *Bifidobacterium* and *Eggerthella* and higher numbers of *Dialister*, *Escherichia-Shigella*, *Fusobacterium*, and *Prevotella* in patients with obesity [12]. Subtle differences were observed in gut microbiota composition between patients with/without obesity, including the differential abundance of genera such as *Escherichia-Shigella*, *Parasutterella*, and *Roseburia*. Notably, enteric bacterial genera, such as *Roseburia*, encode the β-glucuronidase enzyme that deconjugates oestrogens into their active forms in the gut. The collection of bacteria (and their genes) is collectively known as the oestrobolome, which modulates the availability of circulating oestrogens [33]; thus, gut dysbiosis could prompt the differential secretion (and activity) of β-glucuronidase and influence hormonal/metabolic processes to contribute to conditions such as obesity, polycystic ovary syndrome, cancer, endometrial hyperplasia, and endometriosis [21,34]. Therefore, a distinct gut microbiota composition in women with obesity could impact hormone metabolism, displace the window of implantation, and contribute to infertility.

When considering the gut microbiota, significant differences in the B/F ratio between patients with differing BMI values were not observed; therefore, these findings do not support this measure as a hallmark of obesity [26]. Previous studies have provided contrasting results regarding the proportion of Bacteroidetes and Firmicutes in FE samples from patients with obesity [35,36,37]. Our data contribute to this much-debated topic and support a lack of dysbiosis when considering B/F ratios with increasing BMI values.

The two reproductive tract sample types (EF and VA) displayed the lowest alpha diversity values, which agrees with other studies consistently reporting the less diverse nature of genital tract bacterial populations compared with the gut and oral cavity [38]. Additionally, previous reports demonstrated similar EF and VA sample microbiota compositions under healthy and pathological conditions [19,20].

In this study, EF samples were dominated by *Lactobacillus*, although other abundant genera were present (e.g., *Gardnerella*, *Streptococcus*, *Atopobium*, and *Prevotella*). VA samples possessed a microbiota similar to EF samples, with a significant average abundance of *Lactobacillus*, *Gardnerella*, *Prevotella*, and *Streptococcus*. These findings confirm the ascension of vagina-resident bacteria through the cervix as the source of bacterial endometrial colonization [39]; however, endometrial colonisation from alternative routes under particular circumstances cannot be discounted. The EF and VA samples contained additional genera such as *Atopobium*, *Bacteroides*, *Clostridium sensu stricto*, and *Escherichia-Shigella*. Significant differences in vaginal and cervical microbiota compositions in female obesity models have previously been reported [22,23,24]. An association between the presence of bacterial pathogens (e.g., *Atopobium* and *Gardnerella*) and chronic endometritis-causing bacteria (e.g., Enterobacteriaceae, *Staphylococcus*, and *Streptococcus*) with unsuccessful reproductive outcomes was recently discovered [19]. Significant differences between the vaginal or endometrial microbiota in patients with increasing BMI values were not identified; however, a tendency for a pathogenic *Streptococcus*-dominated (>50%) endometrial and vaginal microbiota in patients with obesity was observed. A prospective analysis of 342 endometrial samples from IVF patients found that a higher abundance of Lactobacilli in the endometrium was associated with reproductive success (live birth); however, this study also linked endometrial dysbiosis (including increased levels of *Atopobium*, Enterobacteriaceae, *Gardnerella*, *Haemophilus*, *Klebsiella*, *Staphylococcus*, and *Streptococcus* to the detriment of *Lactobacillus* levels) with reproductive failure (no pregnancy, biochemical pregnancy, and clinical miscarriage) after embryo transfer [19]. Endometrial dysbiosis may cause chronic inflammation, which disrupts the immune balance necessary for embryo implantation; furthermore, the higher prevalence of a *Streptococcus*-dominated endometrial microbiota in women with obesity agrees with the recently reported association between obesity and the prevalence of pathogenic endometrial bacterial profiles (including *Streptococcus*) in women undergoing fertility treatment [40].These findings could help to define why infertile patients with obesity present poorer reproductive outcomes after ART, as this pathogenic genus has been previously linked with repeated implantation failure and clinical miscarriage in infertile patients [19].

Subtle differences were detected between patients with normal weight or obesity; however, a microbiota “fingerprint” of obesity could not be described. As our study cohort comprised only infertile patients, normal weight patients could also possess a dysbiotic microbiota that masks differences. Furthermore, genetics, physical activity, and food intake must be considered as potentially confounding factors when studying the relationship between microbiota and infertility in patients with obesity [41]. Due to the small number of patients, subdivisions of BMI groups could not be performed; thus, a more extensive study population could be crucial in gathering results with substantial statistical power and exploring other associations.

Considering that the bacterial communities of specific body sites have been linked to disease development, defining site-specific compositions of microbiota in infertile women with obesity could help us understand why these patients suffer from poorer reproductive outcomes after ART more often and which lifestyle/therapeutic choices could have a significant influence.

## 4. Materials and Methods

### 4.1. Study Design

This multicentre prospective observational study analysed the endometrial, vaginal, oral, and gut microbiota of infertile patients classified according to BMI values in groups—normal weight (Nw, n = 20, BMI 18.5–29.9 Kg/m^2^) and obesity class I (Ob-I, n = 20, BMI 30.0–34.9 Kg/m^2^), II (Ob-II, n = 20, BMI 35.0–39.9 Kg/m^2^), and III (Ob-III, n = 23, BMI ≥ 40 Kg/m^2^). The number of recruited patients reflects the estimated number of infertile women with obesity seeking ART treatments from four different IVIRMA centres (Spain) within the study period. All samples were collected during the expected window of implantation after 120 h of progesterone administration in a hormone replacement therapy cycle [15]. The bacterial microbiota were analysed by 16S ribosomal (r)RNA gene sequencing. Full description on sample collection and sequencing-based analysis can be found in the Supporting Materials.

### 4.2. Ethical Approval

Ethical approval was given by the local Ethics Committee at the Instituto Valenciano de Infertilidad (Federal Wide Assurance number: FWA00027749) to the 1711-VLC-108-JB protocol. All participants provided written informed consent.

#### Study Population—Inclusion/Exclusion Criteria

From June 2018 to September 2021, 83 participants were recruited from four IVIRMA clinics. Inclusion criteria were infertile patients ≤ 45 years undergoing a hormone replacement therapy cycle for endometrial preparation; negative serological tests for HIV, Hepatitis B/C, and syphilis; and normal uterine morphology assessed by 2D/3D ultrasound (absence of uterine pathology including submucosal/intramural fibroids > 4 cm deforming the cavity, endometrial polyps, Müllerian anomalies, synechiae, or communicating hydrosalpinges). Exclusion criteria included patients with prescribed antibiotics three months before sample collection; carriers of intrauterine devices/oral contraceptives three months before sample collection; the presence of uncorrected uterine pathologies/uncorrected hydrosalpinx; recurrent pregnancy loss (≥2 spontaneous miscarriages); repeated implantation failure (no gestation after transfer of six day-3 embryos or four blastocysts in an in vitro fertilisation or ovum donation cycle); severe or uncontrolled bacterial, fungal/viral infections; and any illness/medical condition representing a risk to patient safety.

### 4.3. Sample Collection

Saliva (SA) and faecal (FE) samples were self-collected by patients using the DNA/RNA Shield™ Saliva Collection Kit (Zymo Research, Irvine, CA, USA) and DNA/RNA Shield™ Fecal Collection Tube (Zymo Research). Vaginal swabs (VA) and endometrial fluid (EF) samples were collected in the clinic by research medical personnel. Vaginal swabs from the posterior fornix were placed in DNA/RNA Shield™ Collection Tube w/Swab (Zymo Research), while EF aspirates were transferred to DNA/RNA Shield™ Collection Tube (Zymo Research). All samples from each patient were collected on the same day. Samples were shipped at room temperature and stored at −80 °C until use. For details, please see the Supporting Information.

### 4.4. DNA Isolation

Total bacterial DNA was extracted using ZymoBIOMICS DNA Miniprep Kits (D4300, Zymo Research). Bacterial DNA was eluted with 50 μL nuclease-free water and sent for library preparation.

### 4.5. 16S Ribosomal RNA Gene Sequencing

Next-generation sequencing obtained bacterial profiles using the Ion 16S Metagenomics kit (Thermo Fisher Scientific, Waltham, MA, USA), following the manufacturer’s instructions. Briefly, after amplifying hypervariable regions with 10 μL of samples (per set of primers) and 30 PCR cycles, the library was prepared from 50 ng pooled short amplicons using the Ion Plus Fragment Library kit and Ion Xpress Barcode Adaptors. The library concentration was adjusted using the Ion Universal Library Quantitation Kit and QuantStudio 5 Real-Time PCR System. The diluted individual libraries were pooled for amplification by emersion PCR in the Ion OneTouch 2 System (10 pM). Finally, libraries were sequenced on the Ion S5 XL system using the Ion 530 Chip (all Thermo Fisher Scientific).

Each sequencing run included 2–4 blank samples and negative and positive PCR controls to detect contamination. Blank samples consisted of sample preservation buffer (DNA/RNA Shield; Zymo Research), while positive and negative PCR controls were pure microbial DNA from *E. coli* (3 ng) and nuclease-free water (Thermo Fisher Scientific).

### 4.6. Sequencing-Based Analysis of Microbiota

16S rRNA sequences were analysed using QIIME 2.0. Quality control and taxonomic assignment were performed using DADA2 and BLAST+ classifiers and the SILVA 132 database, respectively. Data were transformed using the centred log ratio (clr) transformation. Only taxa exhibiting at least 0.1% abundance in all samples were retained [42]. Genera known not to colonise humans/consistently associated with kitome contaminants were removed from the analysis as previously described [19]. For details, please see the Supporting Information.

### 4.7. Statistical Analyses

Numerical variables in groups were described as mean and standard deviation and compared by analyses of variance (ANOVA, normal variables) or Kruskal–Wallis Rank Sum Tests (non-normal variables). Fisher’s exact tests compared categorical variables, described by counts (n) and percentages (%). Pairwise comparisons between groups were conducted when a significant statistical difference was found. For numerical variables, the Pairwise Tukey’s Honestly Significant Difference test or Pairwise Wilcoxon Rank Sum Test with Bonferroni correction accounted for multiple testing, depending on assumptions and distributional data properties. For microbiota analyses, variables were compared using PERMANOVA. *p* values < 0.05 were considered statistically significant.

## 5. Conclusions

The body mass index values correlated with observed variations in gut and reproductive tract microbiota in infertile patients; additionally, decreased levels of *Escherichia-Shigella*, increased *Parasutterella*/*Roseburia*, and a *Streptococcus*-dominated pathogenic endometrial microbiota all have links to obesity. This description of the digestive and reproductive tract microbiota could explain the poorer reproductive outcomes observed in women with obesity. Although the sample size is limited due to the pilot nature of this study, the results provide valuable preliminary insights and support the generation of hypotheses for future research with adequately powered sample sizes.

## Figures and Tables

**Table 1:** Demographic and clinical characteristics of recruited patients and group assignment.

	Overall	Normal Weight	Obese I	Obese II	Obese III	*p* Value
BMI group (Kg/m^2^)		18.5–29.9	30.0–34.9	35.0–39.9	≥40.0	
Number of subjects	83	20	20	20	23	
Age (years, mean (SD))	37.63 (4.30)	36.85 (4.03)	38.15 (4.37)	37.90 (3.78)	37.61 (5.01)	NS
Weight (Kg, mean (SD))	94.93 (20.75)	64.33 (9.46) ^a,b,c^	93.32 (8.17) ^a,d^	103.81 (10.27) ^b^	113.87 (10.25) ^c,d^	<0.001
Height (cm, mean (SD))	165.22 (6.76)	165.20 (6.14)	168.65 (6.07) ^a^	165.50 (7.09)	162.00 (6.41) ^a^	0.013
BMI (Kg/m^2^, mean (SD))	34.42 (7.75)	23.25 (2.54) ^a,b,c^	32.48 (1.63) ^a,d^	37.28 (1.75)^b,e^	43.34 (2.41) ^c,d,e^	<0.001
ART indication, n (%)						NS
AMA	32 (38.6)	7 (35.0)	9 (45.0)	7 (35.0)	9 (39.1)	
Endometriosis	3 (3.6)	2 (10.0)	0 (0.0)	0 (0.0)	1 (4.3)	
Genetic	2 (2.4)	0 (0.0)	1 (5.0)	0 (0.0)	1 (4.3)	
Low ovarian reserve	1 (1.2)	0 (0.0)	0 (0.0)	1 (5.0)	0 (0.0)	
Male factor	15 (18.1)	3 (15.0)	5 (25.0)	3 (15.0)	4 (17.4)	
Oncofertility	1 (1.2)	0 (0.0)	1 (5.0)	0 (0.0)	0 (0.0)	
PCOS	5 (6.0)	2 (10.0)	1 (5.0)	1 (5.0)	1 (4.3)	
IUI failure	2 (2.4)	2 (10.0)	0 (0.0)	0 (0.0)	0 (0.0)	
Single	8 (9.6)	0 (0.0)	2 (10.0)	4 (20.0)	2 (8.7)	
Tubal factor	1 (1.2)	0 (0.0)	0 (0.0)	1 (5.0)	0 (0.0)	
Unknown	13 (15.7)	4 (20.0)	1 (5.0)	3 (15.0)	5 (21.7)	
Time of infertility (years, mean (SD))	2.59 (2.15)	2.67 (2.25)	2.31 (1.78)	2.62 (2.61)	2.73 (2.04)	NS
TSH (mcUI/mL, mean (SD))	2.58 (1.54)	2.60 (1.63)	2.81 (1.95)	2.89 (1.38)	1.89 (1.05)	NS
PRL (ng/mL, mean (SD))	18.20 (10.88)	16.56 (6.56)	16.89 (4.43)	28.31 (23.04)	15.28 (7.32)	NS
Previous pregnancies, n (%)						NS
Yes	16 (19.3)	7 (35.0)	3 (15.0)	3 (15.0)	3 (13.0)	
No	67 (80.7)	13 (65.0)	17 (85.0)	17 (85.0)	20 (87.0)	
Previous biochemical pregnancies, n (%)						NS
Yes	2 (2.4)	0 (0.0)	0 (0.0)	1 (5.0)	1 (4.3)	
No	81 (97.6)	20 (100.0)	20 (100.0)	19 (95.0)	22 (95.7)	
Previous miscarriages, n (%)						NS
Yes	7 (8.4)	3 (15.0)	2 (10.0)	1 (5.0)	1 (4.3)	
No	76 (91.6)	17 (85.0)	18 (90.0)	19 (95.0)	22 (95.7)	
Previous livebirth, n (%)						NS
Yes	8 (9.6)	4 (20.0)	0 (0.0)	2 (10.0)	2 (8.7)	
No	75 (90.4)	16 (80.0)	20 (100.0)	18 (90.0)	21 (91.3)	
Previous ART, n (%)						NS
Yes	39 (47.0)	11 (55.0)	8 (40.0)	8 (40.0)	12 (52.2)	
No	44 (53.0)	9 (45.0)	12 (60.0)	12 (60.0)	11 (47.8)	
PCOS, n (%)						NS
Yes	12 (14.5)	3 (15.0)	4 (20.0)	1 (5.0)	4 (17.4)	
No	69 (83.1)	15 (75.0)	16 (80.0)	19 (95.0)	19 (82.6)	
NA	2 (2.4)	2 (10.0)	0 (0.0)	0 (0.0)	0 (0.0)	
Concomitant pathology, n (%)						NS
Yes	17 (20.5)	4 (20.0)	3 (15.0)	5 (25.0)	5 (21.7)	
No	64 (77.1)	15 (75.0)	17 (85.0)	14 (70.0)	18 (78.3)	
Unknown	2 (2.4)	1 (5.0)	0 (0.0)	1 (5.0)	0 (0.0)	
Concomitant medication, n (%)						NS
Yes	31 (37.3)	4 (20.0)	8 (40.0)	11 (55.0)	8 (34.8)	
No	44 (53.0)	12 (60.0)	10 (50.0)	8 (40.0)	14 (60.9)	
Unknown	8 (9.6)	4 (20.0)	2 (10.0)	1 (5.0)	1 (4.3)	
Endometrial preparation						
E2 intake (days, mean (SD))	10.66 (3.28)	11.78 (3.41)	11.05 (3.95)	10.37 (3.24)	9.64 (2.24)	NS
E2 at day P + 0 (pg/mL, mean (SD))	199.44 (69.86)	215.61 (52.24)	200.00 (76.99)	184.03 (59.80)	201.44 (84.19)	NS
P4 at day P + 0 (ng/mL, mean (SD))	0.55 (2.33)	0.16 (0.14)	0.20 (0.20)	1.11 (4.03)	0.70 (2.27)	NS
Endometrial thickness at P + 0 (mm, mean (SD))	9.40 (1.81)	8.86 (1.24)	9.42 (2.01)	9.14 (1.60)	10.07 (2.09)	NS
E2 at sample collection (pg/mL, mean (SD))	193.70 (74.72)	189.20 (82.43)	169.15 (40.48)	194.75 (81.94)	221.82 (82.50)	NS
P4 at sample collection (ng/mL, mean (SD))	11.00 (5.82)	12.93 (7.19)	11.27 (5.00)	10.18 (4.99)	9.64 (5.84)	NS

Analysis of variance (ANOVA) or Kruskal–Wallis Rank Sum Test were employed to compare numerical variables in the four groups, assuming a two-tailed distribution. ANOVA was used for parametric analysis of normal-distributed variables with homogeneity of variances. Conversely, the Kruskal–Wallis Rank Sum Test was performed as a non-parametric test. Pairwise comparisons between group levels were conducted when a significant statistical difference was found (*p*-value < 0.05). Pairwise group comparisons showing significant differences were labelled using letters (a–e). For numerical variables, the Pairwise Tukey’s Honestly Significant Difference (HSD) test or the Pairwise Wilcoxon Rank Sum Test with Bonferroni correction was employed to account for multiple testing, depending on the assumptions and distributional properties of the data. Abbreviations: AMA, advanced maternal age; ART, assisted reproductive techniques; E2, oestradiol; IUI, intrauterine insemination; NA, not available; NS, not significant; PCOS, polycystic ovarian syndrome; PRL, prolactin; P4, progesterone; P + 0, day of initiation of exogenous P4 in an HRT cycle; SD, standard deviation; TSH, thyroid stimulating hormone.

## Data Availability

The data underlying this article are available in Sequence Read Archives (SRA) at https://www.ncbi.nlm.nih.gov/sra and can be accessed with the BioProject ID PRJNA993585.

## Supplementary Materials

The supporting information can be downloaded at: https://www.mdpi.com/article/10.3390/ijms252312600/s1. References [43,44,45,46,47,48,49,50,51] are cited in the supplementary materials.
