# Supplementary material for: An Analysis of the Digestive and Reproductive Tract Microbiota in Infertile Women with Obesity
Source: Int J Mol Sci. 2024 Nov 23;25(23):12600. doi: 10.3390/ijms252312600 (PMC11641297; doi:10.3390/ijms252312600)
Supplement: Supplementary file 1 [file ijms-25-12600-s001.zip › Supporting Methods.pdf]

**SUPPORTING METHODS - An Analysis of the Digestive and Reproductive Tract Microbiota in Infertile Women with Obesity**

**Sample collection**

*Saliva (SA) samples* were self-collected using the DNA/RNA Shield™ Saliva Collection Kit (Zymo Research, CA, USA). Patients were asked not to eat solid/liquid food for 2 h before sample collection; patients then rinsed their mouths with water and directly deposited SA samples into tubes containing 2 mL preserving solution.

Patients collected *fecal (FE) sample*s at home on the same day they provided other samples in provided tubes (DNA/RNA Shield™ Fecal Collection Tube, Zymo Research, CA, USA) containing 9 mL preserving solution.

*Vaginal (VA) and endometrial fluid (EF) samples* were collected in the clinic by research medical personnel. With the patient in the lithotomy position, the external vagina was cleansed after introducing the disinfected speculum and before obtaining the endometrial fluid aspirate. The posterior fornix was swabbed to collect vaginal discharge. The swab was placed in a DNA/RNA Shield™ Collection Tube w/Swab (Zymo Research, CA, USA) containing 1 mL preserving solution. After collecting the vaginal swab, the cervix was cleaned with sterile gauze. After aspiration of the cervical mucus to avoid contamination, a sterile, flexible embryo transfer catheter was inserted, and EF was aspirated (20-40 µl with a 10-mL syringe). Contact with the vaginal walls was avoided to prevent contamination, and suction was stopped at the entrance of the internal cervical os during catheter removal. The EF was transferred to a DNA/RNA Shield™ Collection Tube (Zymo Research, CA, USA) containing 0.1 mL preserving solution.

All samples were shipped at room temperature and stored at −80ºC until use.

**Sequencing-based analysis of microbiota**

16S rRNA sequencing results were analyzed using the QIIME 2.0 package [1]. Quality control and denoising of sequences were conducted using the DADA2 algorithm [2], with BLAST+ classifier [3] for taxonomic assignment, along with the SILVA 132 database [4]. The BLAST+ classifier performs a local alignment between query and reference reads for processed samples. Consensus taxonomy was assigned to each query sequence. The results were generated using default parameters in QIIME 2.0 and BLAST+. Identification/removal of contaminant sequences was performed using the decontam R Bioconductor package [5] using bacterial prevalence in blank samples to statistically identify contaminating taxa. Data were transformed using the centered log ratio (clr) transformation [6], making them symmetric and linearly related, thus avoiding spurious correlations and sub-compositional incoherencies [7–9]. The abundance of each taxon was analyzed, and low-abundance species were removed.

**References:**

1. Bolyen E, Rideout JR, Dillon MR, *et al.* Reproducible, interactive, scalable and extensible microbiome data science using QIIME 2. *Nat Biotechnol* 2019;37:852–857.

2. Callahan BJ, McMurdie PJ, Rosen MJ, Han AW, Johnson AJA, Holmes SP. DADA2: High-resolution sample inference from Illumina amplicon data. *Nat Methods* 2016;13:581–583.

3. Camacho C, Coulouris G, Avagyan V, *et al.* BLAST+: architecture and applications. *BMC Bioinformatics* 2009;10:421.

4. Quast C, Pruesse E, Yilmaz P, *et al.* The SILVA ribosomal RNA gene database project: improved data processing and web-based tools. *Nucleic Acids Res* 2012;41:D590–D596.

5. Davis NM, Proctor DM, Holmes SP, Relman DA, Callahan BJ. Simple statistical identification and removal of contaminant sequences in marker-gene and metagenomics data. *Microbiome* 2018;6:226.

6. Aitchison J. *The Statistical Analysis of Compositional Data*. Chapman & Hall; 1986.

7. Calle ML. Statistical Analysis of Metagenomics Data. *Genomics Inform* 2019;17:e6.

8. Gloor GB, Macklaim JM, Pawlowsky-Glahn V, Egozcue JJ. Microbiome Datasets Are Compositional: And This Is Not Optional. *Front Microbiol* 2017;8:2224.

9. Pawlowsky-Glahn V, Egozcue JJ, Tolosana-Delgado R. *Modeling and analysis of compositional data*. John Wiley & Sons; 2015.
