# Supplementary material for: An Analysis of the Digestive and Reproductive Tract Microbiota in Infertile Women with Obesity
Source: Int J Mol Sci. 2024 Nov 23;25(23):12600. doi: 10.3390/ijms252312600 (PMC11641297; doi:10.3390/ijms252312600)
Supplement: Supplementary file 1 [file ijms-25-12600-s001.zip › TableS1.pdf]

Table S1. Relative abundance of chronic endometritis-associated bacteria in normoweight and infertile patients with obesity.

**Sample type**	**Endometrial Fluid**				**Vaginal Aspirate**			
**BMI group (Kg/m^2)**	**Normoweight**<br>**18.5-29.9**	**Obese I**<br>**30.0-34.9**	**Obese II**<br>**35.0-39.9**	**Obese III**<br>**≥40.0**	**Normoweight**<br>**18.5-29.9**	**Obese I**<br>**30.0-34.9**	**Obese II**<br>**35.0-39.9**	**Obese III**<br>**≥40.0**
**Total number of samples (n)**	19	20	19	22	20	20	20	22
Escherichia-Shigella								
Average abundance (%)	**1.43**	**0**	**0**	**2.45**	**1.61**	**1.52**	**0**	**4.61**
Presence (n)	1	0	0	3	1	1	0	3
Dominance (n)	0	0	0	0	0	0	0	0
Gardnerella								
Average abundance (%)	**9.50**	**3.91**	**8.40**	**2.60**	**10.65**	**3.82**	**10.80**	**5.36**
Presence (n)	8	5	9	5	9	6	8	4
Dominance (n)	0	0	0	0	0	0	0	0
Klebsiella								
Average abundance (%)	**0**	**0**	**0**	**0**	**0**	**1.39**	**0**	**0**
Presence (n)	0	0	0	0	0	1	0	0
Dominance (n)	0	0	0	0	0	0	0	0
Neisseria								

Average abundance (%)	**1.40**	**1.14**	**0**	**0**	**0**	**0**	**0**	**0**
Presence (n)	1	1	0	0	0	0	0	0
Dominance (n)	0	0	0	0	0	0	0	0
Streptococcus								
Average abundance (%)	**5.92**	**12.63**	**23.26**	**18.29**	**13.38**	**19.60**	**56.37**	**29.19**
Presence (n)	5	6	7	6	3	3	7	4
Dominance (n)	0	2	1	1	0	1	1	0
Ureaplasma								
Average abundance (%)	**1.53**	**1.28**	**0**	**0**	**2.89**	**3.18**	**1.92**	**1.44**
Presence (n)	1	1	0	0	1	2	2	1
Dominance (n)	0	0	0	0	0	0	0	0

Abbreviations: BMI, body mass index; **EF**, endometrial fluid; **VA**, vagina. **Average abundance** is calculated only for those patients with presence of each genus (over 1%); **Presence**, number of patients with relative abundance >1%; **Dominance**, number of patients with relative abundance >50%.
