# Supplementary figures and images for: An Analysis of the Digestive and Reproductive Tract Microbiota in Infertile Women with Obesity
Source: Int J Mol Sci. 2024 Nov 23;25(23):12600. doi: 10.3390/ijms252312600 (PMC11641297; doi:10.3390/ijms252312600)

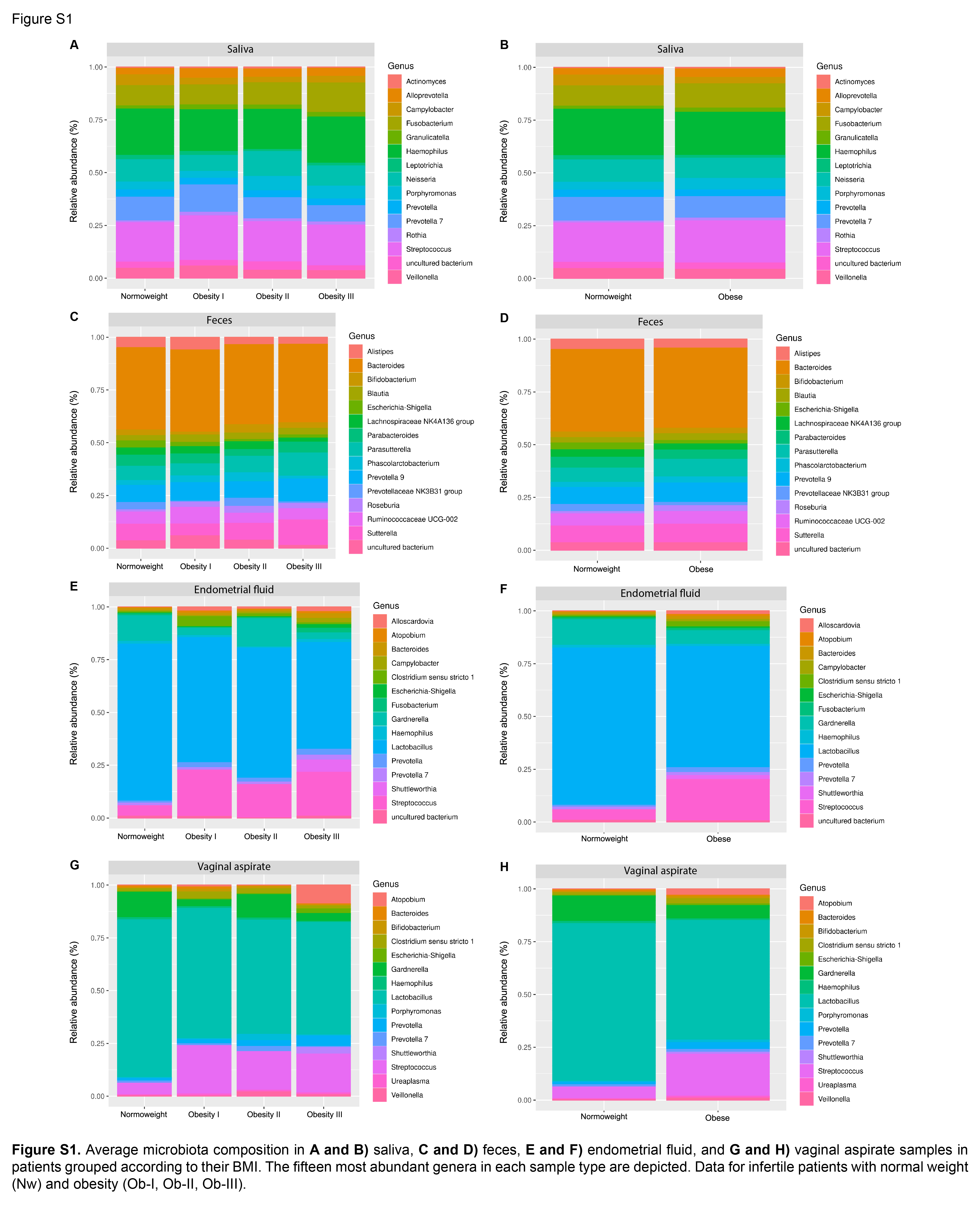

Supplement: Supplementary file 1 [file ijms-25-12600-s001.zip › Figure S1.jpg]

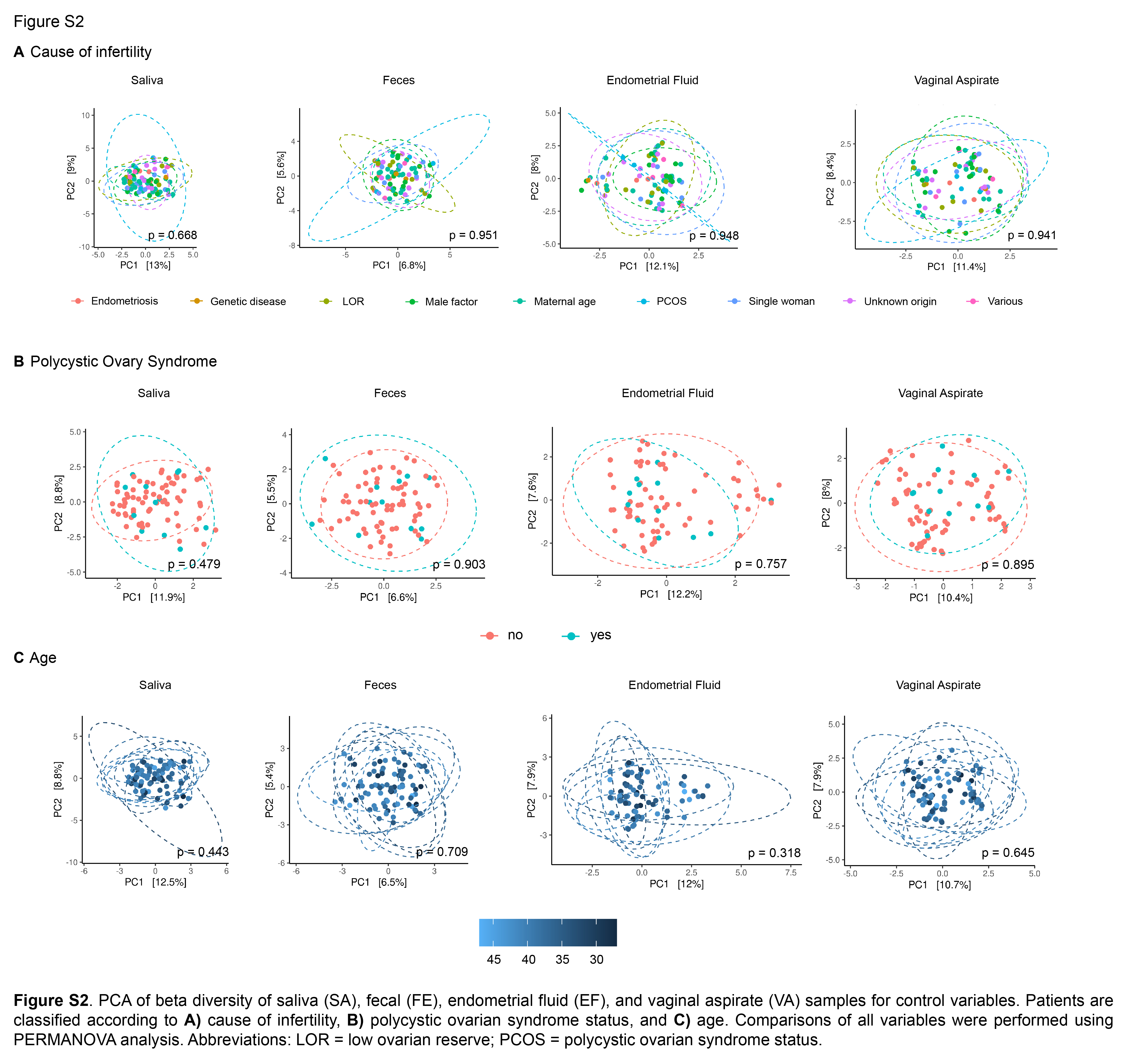

Supplement: Supplementary file 1 [file ijms-25-12600-s001.zip › Figure S2.jpg]
